# Revisiting “An Exercise in Groundwater Model Calibration and Prediction” After 30 Years: Insights and New Directions

**DOI:** 10.1111/gwat.12907

**Published:** 2019-07-02

**Authors:** Randall J. Hunt, Michael N. Fienen, Jeremy T. White

**Affiliations:** ^1^ U.S. Geological Survey, Upper Midwest Water Science Center, 8505 Research Way Middleton Wisconsin 53562; ^2^ Institute of Geological and Nuclear Sciences, Limited, Wairakei Research Centre, 114 Karetoto Road, RD4, Taupo, 3384 New Zealand

## Abstract

In 1988, an important publication moved model calibration and forecasting beyond case studies and theoretical analysis. It reported on a somewhat idyllic graduate student modeling exercise where many of the system properties were known; the primary forecasts of interest were heads in pumping wells after a river was modified. The model was calibrated using manual trial‐and‐error approaches where a model's forecast quality was not related to how well it was calibrated. Here, we investigate whether tools widely available today obviate the shortcomings identified 30 years ago. A reconstructed version of the 1988 true model was tested using increasing parameter estimation sophistication. The parameter estimation demonstrated the inverse problem was non‐unique because only head data were available for calibration. When a flux observation was included, current parameter estimation approaches were able to overcome all calibration and forecast issues noted in 1988. The best forecasts were obtained from a highly parameterized model that used pilot points for hydraulic conductivity and was constrained with soft knowledge. Like the 1988 results, however, the best calibrated model did not produce the best forecasts due to parameter overfitting. Finally, a computationally frugal linear uncertainty analysis demonstrated that the single‐zone model was oversimplified, with only half of the forecasts falling within the calculated uncertainty bounds. Uncertainties from the highly parameterized models had all six forecasts within the calculated uncertainty. The current results outperformed those of the 1988 effort, demonstrating the value of quantitative parameter estimation and uncertainty analysis methods.

## Abstract 2

*Article impact statement*: The power of today's calibration and uncertainty tools and approaches is demonstrated by revisiting a landmark work from 30 years ago.

## Introduction

Thirty years ago, Freyberg (1988) documented an exercise where nine groups of graduate students calibrated a groundwater model and then used it to make forecasts. The intent was to provide a perspective different from that of case studies and theoretical investigations. The problem was considered somewhat idyllic: the numerical model system geometry, discretization, and boundary conditions given the students was correct for the system, and observations used for calibration were error‐free. The students were directed to “calibrate” the model and then a (deterministic) forecast was made under different boundary conditions. Yet, a limited evaluation of the resulting calibrated models and the associated forecasts showed that: (1) there were significant differences among the groups with respect to calibration strategies, parameterizations, and associated forecasts; and (2) the quality of a forecast was unrelated to the quality of calibration.

These results influenced a generation of modelers. They underscored the deficiencies of “point calibration,” where hydraulic conductivity (*K*) is locally varied around observations solely to obtain a better fit, even at the expense of geological realism. The results also called into question the ability to make forecasts even for cases of relatively small modifications to base case conditions. Less obviously, it exposed the intractable shortcomings of limiting calibration to manual trial‐and‐error where there is no quantitative or systematic evaluation, and details about how each group obtained their results were only narrative. These observations formed the motivation to use this problem in a parameter estimation course we teach and prompted revisitation of the original problem. That is: (1) could calibration and uncertainty analysis tools now widely available obviate the shortcomings identified by Freyberg (1988); and (2) if so, could the insights of Freyberg (1988) be updated to reflect what we have learned in the 30 years since it was published?

### Presentation Approach and Problem Description

To make the original Freyberg problem, and insights presented here, more accessible for the reader, model files are provided online (Hunt et al. 2019). We use a stepwise approach so that the tools brought to bear generally increase in sophistication. A detailed description of the original problem given to the students is included on pages 351‐356 of Freyberg (1988); the main characteristics of the problem are briefly relayed here. The description included two parts: (a) one involving the hypothetical aquifer system; and (b) one focusing on calibration and forecasting goals and metrics for success. For brevity, detailed explanation of the history of parameter estimation and its tools are not discussed here, but is available from others (e.g., page 397 in Anderson et al. 2015; Zhou et al. 2014, respectively).

#### *Hypothetical Aquifer System*

**Figure 1:**
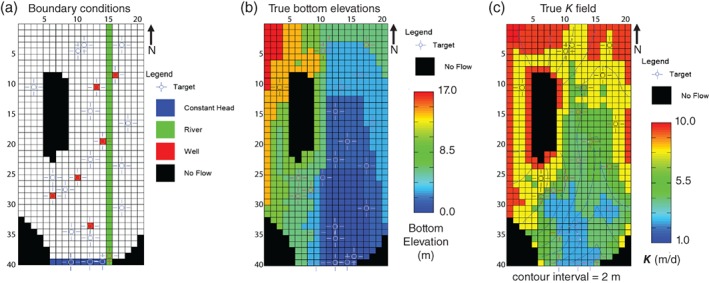
Boundary conditions, observation locations (a), true bottom elevations (b), true hydraulic conductivity (*K*) field, and simulated water table (c) for the recreated Freyberg (1988) model.

MODFLOW‐2005 (Harbaugh et al. 2017) was used to simulate a single‐layer, shallow, water‐table aquifer surrounded with no‐flow boundaries on the bottom and north‐east‐west sides and a specified head boundary on the southern boundary (Figure 1a). The MODFLOW grid had 40 rows and 20 columns (250 m on a side), inactive outcrop areas within the grid, and a straight river through the extent of the model domain in column 15. There were six pumping wells and spatially uniform recharge rate (*R)*. All aquifer stresses and boundary conditions were constant in time (steady‐state). The bottom elevations were not uniform; the aquifer was relatively flat on the east side and sloped gently to the south and west sides (Figure 1b). In the western area, and southeastern and southwestern corners of the aquifer, the impermeable bottom elevation was higher making for no‐flow outcrop areas within the grid. Hydraulic conductivity (*K*) consisted of six zones (Figure 1c) with relatively small changes among them; areas of higher values of *K* were along the north, east and west boundaries, and adjacent to the western outcrop; *K* was lower in the south. A range of values were reported for the zones (e.g., Freyberg 1988, Figure 3); the original model input was not available, so the midpoint of the zone range was assigned the “true” value for each zone (resulting *K* range = 2.6 to 10.4 m/d). The water table and water budget MODFLOW calculated for this synthetic true model is shown in Figure 1c and Table 1, respectively. Notable features of the true model included: effect of the river and pumping are expressed in the water table, and a groundwater divide occurred in the area between the outcrop and the western no‐flow boundary (Figure 1c).

**Table 1:** Comparison of 2018 Recreated Model to 1988 True Model Fluxes

	2018 Recreation	1988 True Model
	(×10^−3^ m^3^/s)	(×10^−3^ m^3^/s)
IN		
STORAGE	0	0
CONSTANT HEAD	0	0
WELLS	0	0
RIVER LEAKAGE	1.58	1.53
RECHARGE	69.38	69.5
TOTAL IN	70.96	71.00
OUT		
STORAGE	0	0
CONSTANT HEAD	4.39	4.95
WELLS	22.05	22.05
RIVER LEAKAGE	44.52	44.00
RECHARGE	0	0
TOTAL OUT	70.96	71.00

### Calibration and Forecast Objectives

Freyberg (1988) states, “The information available to the participants was, in fact, quite extensive [they] knew: the exact horizontal geometry of the aquifer, including discretization; the nature of the boundary conditions; all information characterizing the interaction of the river with the aquifer; and all production well pumping rates.” Only a subset of the data was available, however, for heads and aquifer thickness. Sixteen observation wells were provided (“targets” on Figure 1), and aquifer thickness was only known at those locations and the pumping wells. The purpose of the exercise was to forecast the change in head at the six pumping wells resulting from lining the river channel (simulated by reducing the river conductance by 2 orders of magnitude).

The metrics of success given the students were the root mean square error of observed‐minus‐simulated forecasted future head at the six pumping wells, the root mean square error difference between estimated and true *K* for all 705 active model cells, and the number of calibration model runs. The participants' success did not depend on how well they fit the observed head data, yet they had to “develop a calibration measure for premodification conditions that would lead to a good estimation of the conductivity field and to a good prediction of the aquifer water‐table elevations under modified conditions” (page 356 in Freyberg 1988). Presumably, each of the nine student groups employed some type of history matching between the simulated and observed heads in the expectation that reproducing past conditions improves the ability to the model to forecast future outcomes—a fundamental concept in applied groundwater modeling.

### Application of Parameter Estimation and Uncertainty Tools

We used this model description from Freyberg (1988) to reconstruct the “true” model. Once the truth was reconstructed, we simplified the model to reflect unknowns similar to those that the students faced. Specifically, we modified the synthetic truth by:

1. Simplification of the aquifer bottom: The students were given bottom elevations at the pumping and observations wells. Here, the true bottom elevations were disregarded and set to values provided in the 16 cells containing observation and pumping wells and, for all other cells, to the average of the 16 bottom elevations given to the students (average = 3.49 m). We later tested a bottom elevation kriged from given elevations.
2. Simplification of the aquifer *K*: We discarded the six zones used for the true *K* field and initially set the aquifer to the simplest case of one homogeneous zone; we explored additional *K* parameter flexibility during calibration.

The students were told that the recharge rate (*R*) was homogeneous but were not given the “true” value *R*; thus, we parameterized *R* as a single zone. We translated model input and output files for use by the parameter estimation code software suite—PEST (Doherty 2018) and PEST++ suite (Welter et al. 2015)—following the groundwater model calibration guidelines of Doherty and Hunt (2010). We assumed the students did not know the true observation measurement error, (i.e., that in fact the observations were error‐free). Therefore, we assigned an error typical of a head observation to each of the 16 head observations (a standard deviation of 1 m, which results in a PEST weight of 1/standard deviation, or 1 m^−1^). In keeping with Freyberg (1988), we retained the models' ability to simulate the six forecasts of interest as a primary metric for evaluation.

## Results and Discussion

By definition, the root mean square error (RMSE) for the true model was 0.0 for both head calibration and forecasts. Subsequently, the aquifer base and *K* simplifications of the recreated model degraded model calibration and forecast quality (Table 2). We used the model with both the simplified aquifer base and *K* field for subsequent testing. Because parameter estimation has long been called a “necessary next step” for groundwater modeling (Poeter and Hill 1997), it formed the basis for our initial evaluations, which began with simple methods and moved to more sophistication methods. We conclude with an uncertainty analysis—a logical endpoint for models used for decision making.

**Table 2:** Results of Simplifying the True Model

a) Base Elevation Simplification		
True *R* = 1.38 × 10^−4^ m/d		
True *K* field is 6 zones ranging from 2.6 to 10.4 m/d		
Base elevation = 3.34 m (average of 16 elevations)		
	**RMSE**	**RMSE**
	**calibration (m)**	**forecasts (m)**
	0.60	0.17
Freyberg (1988)	0.06–0.37	1.26–7.96
**b) Base Elevation and *K* Simplification**		
True *R* = 1.38 × 10^−4^ m/d		
*K* is 1 zone = 6.7 m/d (average of zone true values)		
Base elevation = 3.34 m (average of 16 elevations)		
	**RMSE**	**RMSE**
	**calibration (m)**	**forecasts (m)**
	0.92	1.23
Freyberg (1988)	0.06–0.37	1.26–7.96

Note(s): Average of true base elevations is 5.41 m (*R* = recharge rate; *K* = horizontal hydraulic conductivity; RMSE = root mean square error; m = meter; d = day).

### Simplest Parameter Estimation

Even though the parameter estimation problem used a well‐constrained system with error‐free head observations, the problem could not be uniquely solved (Table 3). This is evidenced by obtaining similar calibration summary statistics with different parameter sets, where the different optimal parameters resulted from different initial parameter values. Nor are any of the results near the mean of the true *K* field (6.69 m/d) and true *R* (1.38 × 10^−4^ m/d).

**Table 3:** Results of Ill‐Posed Parameter Estimation on the 1 Zone Model

a) Starting Heads Too High					b) Starting Heads Too Low			
1 Zone starting *R* = 5.00 × 10^−4^					1 Zone starting *R* = 1.30 × 10^−4^			
1 Zone starting *K* = 1 m/d					1 Zone starting *K* = 10 m/d			
Base elevation = 3.34 fixed					Base elevation = 3.34 fixed			
		**RMSE**	**RMSE**				**RMSE**	**RMSE**
***K***	***R***	**Calibration**	**Forecasts**		***K***	***R***	**Calibration**	**Forecasts**
**(m/d)**	**(m/d)**	**(m)**	**(m)**		**(m/d)**	**(m/d)**	**(m)**	**(m)**
13.17	3.192 × 10^−4^	0.48	4.11		12.70	3.084 × 10^−4^	0.48	3.92
**c) Starting Heads Too High**					**d) Starting Heads Too Low**			
1 Zone starting *R* = 5.00 × 10^−4^					1 Zone starting *R* = 1.30 × 10^−4^			
1 Zone starting *K* = 4 m/d					1 Zone starting *K* = 20 m/d			
Base elevation = 3.34 fixed					Base elevation = 3.34 fixed			
		**RMSE**	**RMSE**				**RMSE**	**RMSE**
***K***	***R***	**Calibration**	**Forecasts**		***K***	***R***	**Calibration**	**Forecasts**
**(m/d)**	**(m/d)**	**(m)**	**(m)**		**(m/d)**	**(m/d)**	**(m)**	**(m)**
9.40	2.344 × 10^−4^	0.49	2.52		10.71	2.632 × 10^−4^	0.48	3.09

Note(s): Results obtained using PEST v15.0 (R = recharge rate; K = horizontal hydraulic conductivity; RMSE = root mean squared error; m = meter; d = day).

The correlation coefficient between *R* and *K* parameters was over 0.99, as expected, demonstrating the problem is ill‐posed. Given that a 1‐layer model is solved in terms of transmissivity, the additional degree of freedom gained by making the base elevation adjustable would only exacerbate the ill‐posedness. Although the fit was similar across the parameter estimation tries in Table 3 (RMSE calibration), it was worse than the 1988 results. The ability of the “calibrated” model to make good forecasts for the system also varied appreciably (RMSE forecasts) and did not equal the best obtained by the 1988 manual calibration (1.26 m; Table 3).

The inability of head‐only observations to constrain both *R* and transmissivity is well‐known (e.g., page 34 in Hunt 1987; Poeter and Hill 1997; Haitjema 2006), and the recommended practice for groundwater modeling is to use both head and flux observation types (Anderson et al. 2015) to break the correlation. Given the range of results in Table 3, it is clear the quantitative framework of parameter estimation did not make an intractable problem uniquely solvable—in this way, we are no better off than the student groups of 1988! Unlike the work of the student groups of 1988, however, our parameter estimation approach unequivocally documents the ill‐posed and unsolvable nature of the problem (e.g., correlation coefficient >0.99), and underscores that no single correct answer exists given the information provided to the students.

### Advanced Parameter Estimation

The parameter estimation techniques used in 1990s modeling were developed primarily for mathematically “well posed” or “overdetermined” problems—that is, those where there is a unique set of parameters that yield a single, well‐defined, stable, and best‐fit solution to the parameter estimation problem (e.g., Hadamard 1902). Some modelers maintain that a parameter estimation problem is overdetermined when the number of observations is larger than the number of parameters. Accordingly, our version of the Freyberg (1988) problem should be overdetermined: there are two parameters (one *K* and one *R* parameter) and 16 observations. Yet, this problem was decidedly non‐unique (Table 3). A more representative way to assess whether a unique solution exists for the parameter estimation problem is to consider the types of information contained in the observations, and how it might constrain parameters. In this case, 16 head observations help inform the spatial distribution of transmissivity but, by themselves, cannot break the correlation between the *R* and *K* parameters, and this will be the case no matter how many head observations are included.

The problem of non‐uniqueness facing Freyberg's students is, in fact, an inherent issue with all models of the unknowably complex natural world. We will never have enough observations to uniquely inform all parameters used to construct realistic environmental models. Thus, our calibration endeavors are expected to be ill posed (Hunt et al. 2007; Chapter 9 in Anderson et al. 2015). In recognition of the need to solve problems from the real world, and inability of overdetermined methods to do so, approaches were adapted from other fields and refined (described in detail by others, e.g., Doherty and Hunt 2010; Aster et al. 2013; Anderson et al. 2015, among others). Briefly, three widely used methods for obtaining a conditionally unique solution to an ill‐posed parameter estimation problem are: (1) Tikhonov regularization (Tikhonov and Arsenin 1977), where qualitative or soft knowledge about the system is used to inform parameter values; (2) subspace methods, where the effective parameter dimensionality of the problem is reduced through mathematical techniques (e.g., singular value decomposition—SVD), essentially “locking in” any nonunique parameter correlation at the ratios defined by the initial parameter values; and (3) obtaining additional data that can inform the problem differently.

In practice the first two are usually used in combination, where subspace methods create an unconditionally well‐posed problem and the soft‐knowledge reigns in unrealistic parameter values and spatial distributions. Anderson et al. (2015) provides an accessible discussion of these methods for handling ill‐posed problems in groundwater models, and Doherty and Hunt (2010) discussed mechanics and recommended settings for the PEST (Doherty 2018) and PEST++ (Welter et al. 2015) software. Invoking SVD to reduce effective parameter dimensionality and stabilize the inverse problem requires very little input from the modeler; in PEST++, SVD is invoked automatically in recognition that most real‐world models with realistic and representative parameterization are ill‐posed. The results in Table 3, however, were obtained using the PEST software that does not automatically invoke SVD.

The companion strategy of bringing soft‐knowledge to bear through Tikhonov regularization, however, requires more thought from the user. Soft knowledge is qualitative and subjective, therefore the modeler must create inputs that represent parameter conditions they prefer (e.g., preferred value, difference or homogeneity), which parameters to apply them to, what values to prefer, and at what “strength” to apply this soft knowledge. The “strength” informs the tradeoff between the penalty for departing from the preferred conditions and the benefit of a better fit to observations. Returning to the Freyberg (1988) model, what soft knowledge might have been available to the 1988 student groups? They were given pumping rates and heads in observation wells; suppose one pumping well and an adjacent observation well were simplified to a single well pumping test analysis? Suppose further the selected pumping well was the one farthest from the river (row 28, column 6) to minimize potential confounding effects of a recharge boundary. Presumably such an analysis would result in a *K* of 5‐6 m/d because the true *K* was 5.62 m/d. The student would not know how widely the locally derived pumping test value applies, therefore it is better treated as soft knowledge about the aquifer for parameter estimation purposes, implemented as a preferred value where deviations from this preferred value are penalized.

Applying a soft knowledge preferred value of 5.0 m/d for all *K* values in the parameter estimation process yielded a conditionally unique solution to the parameter estimation process (Table 4). Although the oversimplification of the single *K* zone parameterization did not equal the 1988 manual trial‐and‐error head calibration statistics of the students, for forecasts it performed better (lower forecast RMSE in Table 4). In addition, the number of runs required for the parameter estimation is in the range reported by the student groups. Although gratifying, determining a unique “best model” solely through soft‐knowledge is not ideal.

**Table 4:** Results of Simple Soft‐Knowledge Parameter Estimation on the 1 Zone Model

a) Starting Heads Too High					
1 Zone starting *R* = 5.00 × 10^−4^					
1 Zone starting *K* = 1 m/d					
Base elevation = 3.34 fixed					
			**RMSE**	**RMSE**	
**Results**	***K***	***R***	**Calibration**	**Forecasts**	**Number**
**Year**	**(m/d)**	**(m/d)**	**(m)**	**(m)**	**of runs**
2018	5.00	1.313 × 10^−4^	0.54	0.98	37
1988	2.6–10.4	1.380 × 10^−4^	0.06–0.37	1.26–7.96	15–38
**b) Starting Heads Too Low**					
1 Zone starting *R* = 1.30 × 10^−4^					
1 Zone starting *K* = 10 m/d					
Base elevation = 3.34 fixed					
			**RMSE**	**RMSE**	
**Results**	***K***	***R***	**Calibration**	**Forecasts**	**Number**
**Year**	**(m/d)**	**(m/d)**	**(m)**	**(m)**	**of runs**
2018	5.00	1.312 × 10^−4^	0.54	0.98	21
1988	2.6–10.4	1.380 × 10^−4^	0.06–0.37	1.26–7.96	15–38

Note(s): Preferred K value used in Tikhonov regularization = 5.0 m/d; Results obtained using PEST v15.0 (R = recharge rate; K = horizontal hydraulic conductivity; RMSE = root mean squared error; m = meter; d = day).

### Parameter Estimation Using Recommended Approaches

As has been alluded to, the fundamental shortcoming of the 1988 problem was that only head data were available to the students to constrain both *K* and *R*. Hunt and Welter (2010) point out that an underused aspect of models is to assess the value of future data collection (e.g., see James et al. 2009; Dausman et al. 2010; Fienen et al. 2011). As a somewhat trivial example of this ability, we used the model to perform a simple experiment related to the worth of yet‐to‐be‐collected observations by adding a flux target to meet the Anderson et al. (2015) minimum requirement of both head and flux target types. One flux observation was added to the version of the parameter estimation problem without the Tikhonov soft knowledge included (i.e., the model from Table 3).

The flux target was located near the most downgradient edge of model domain (thus encompassing a larger portion of the model domain) and was given an “observed” value (3457.0 m^3^/d) obtained from the previous run where only soft knowledge was used to obtain a conditionally unique solution (i.e., the model of Table 4). The weight of this new observation was calculated assuming coefficient of variation (CV) equal to 0.10; this CV results in a 95% confidence interval spanning approximately ±20%, or ± 2 standard deviations, around the “observed” value calculated by the model of Table 4. The standard deviation equals the CV (0.10) times the specified flux value (3457.0 m^3^/d), or 345.7 m^3^/d. The PEST weight then becomes 1/standard deviation, or 0.0029 d/m^3^. All aspects of the 1988 problem were retained except for inclusion of this one additional flux observation.

As expected, the addition of the flux observation broke the correlation of the *R* and *K* parameters (correlation coefficient of 0.72), which resulted in a conditionally unique solution to the parameter estimation (Table 5a). Optimal *K* and *R* parameter values in Table 5a are near but not exactly those reported in Table 4. Although the model from Table 4 provided the “observed” flux, an identical result is not expected because the weight associated with the new flux observation was not overly high thus not a primary focus of the parameter estimation. Regardless, this formulation of the parameter estimation problem is superior because an observation (“hard data”) facilitated a conditionally unique solution rather than soft knowledge, as was the case for the results in Table 4.

**Table 5:** Results of Adding a Flux Target to the Simple Parameter Estimation of the 1 Zone Model, No Soft‐Knowledge Regularization

a) Observed Flux = 3457.0 m^3^/d Obtained From Soft‐Knowledge‐Only Model of Table 3					
**Starting Heads Too High**					
1 Zone starting *R* = 5.00 × 10^−4^					
1 Zone starting *K* = 1 m/d					
Base elevation = 3.34 fixed					
			**RMSE**	**RMSE**	
**Results**	***K***	***R***	**Calibration**	**Forecasts**	**Number**
**Year**	**(m/d)**	**(m/d)**	**(m)**	**(m)**	**of runs**
2018	5.08	1.322 × 10^−4^	0.54	0.96	33
1988	2.6–10.4	1.380 × 10^−4^	0.06–0.37	1.26–7.96	15–38
**Starting Heads Too Low**					
1 Zone starting *R* = 1.30 × 10^−4^					
1 Zone: starting *K* = 10 m/d					
Base elevation = 3.34 fixed					
			**RMSE**	**RMSE**	
**Results**	***K***	***R***	**Calibration**	**Forecasts**	**Number**
**Year**	**(m/d)**	**(m/d)**	**(m)**	**(m)**	**of runs**
2018	5.08	1.322 × 10^−4^	0.54	0.96	27
1988	2.6–10.4	1.380 × 10^−4^	0.06–0.37	1.26–7.96	15–38
**b) Observed Flux = 3710.5 m^3^/d Obtained from True Model of Table 1**					
**Starting Heads Too High**					
1 Zone starting *R* = 5.00 × 10^−4^					
1 Zone starting *K* = 1 m/d					
Base elevation = 3.34 fixed					
			**RMSE**	**RMSE**	
**Results**	***K***	***R***	**Calibration**	**Forecasts**	**Number**
**Year**	**(m/d)**	**(m/d)**	**(m)**	**(m)**	**of runs**
2018	5.35	1.385 × 10^−4^	0.53	0.88	29
1988	2.6–10.4	1.380 × 10^−4^	0.06–0.37	1.26–7.96	15–38
**Starting Heads Too Low**					
1 Zone starting *R* = 1.30 × 10^−4^					
1 Zone: starting *K* = 10 m/d					
Base elevation = 3.34 fixed					
			**RMSE**	**RMSE**	
**Results**	***K***	***R***	**Calibration**	**Forecasts**	**Number**
**Year**	**(m/d)**	**(m/d)**	**(m)**	**(m)**	**of runs**
2018	5.35	1.385 × 10^−4^	0.53	0.88	27
1988	2.6–10.4	1.380 × 10^−4^	0.06–0.37	1.26–7.96	15–38

Note(s): Tikhonov regularization not used; results obtained using PEST v15.0 (R = recharge rate; K = horizontal hydraulic conductivity; RMSE = root mean squared error; m = meter; d = day).

Having demonstrated the utility of a flux observation for breaking the correlation between *R* and K, we complete our revisit of the 1988 problem now assuming the students were given a flux measurement at this location and the value obtained equaled the MODFLOW simulated value near the true value (3710.5 m^3^/d). To be clear: this flux observation is knowledge that *was not available* to the 1988 student. Therefore, the following comparisons provided are simply for evaluating how modern parameter estimation approaches (using the recommended minimum dataset of head‐and‐flux‐calibration‐target types) fares using metrics given the student groups in 1988.

When the true flux value is included, a conditionally unique solution is obtained (Table 5b)—one with better forecasts than the soft‐knowledge only regularized results (Table 4), and those obtained during the 1988 manual trial‐and‐error calibration (Table 5b), even though the aquifer *K* is represented by one zone. The optimal *R* (1.385 × 10^−4^ m/d) is within 0.2% of the true model (1.3824 × 10^−4^ m/d), but the optimal *K* value (5.35 m/d) was nearly 20% less than the mean of the true *K* field (6.69 m/d). The latter result is due to differences in our base elevation calculated from the 16 values given to the students (mean of the 16 wells = 3.34 m) from true bottom elevations (mean of all bottom nodes in the model domain = 5.38 m), which affect the aquifer transmissivity more than the overall model water balance.

Although uniquely solved, elements of the problem are obviously oversimplified. Most notably, natural aquifers are typically not well represented by a single value of *K*. Oversimplification of a model can be just as detrimental to its forecasts as the widely recognized issue of being too complex (Hunt et al. 2007; Doherty and Christensen 2011). Worse, inappropriate parameter simplification can lead to non‐trivial and undetectable forecast biases (White et al. 2014) because overly simple models limit the ability of information residing within the observations to inform and constrain the parameters in appropriate and meaningful ways. In our model, the use of one *K* zone for 16 head observations results in a parameter estimation problem where the observations can “speak” (inform parameters) but the model does not have the correct means to “hear” (Doherty and Hunt 2009). Thus, another parameter estimation was performed using the recommended highly parameterized approach of Doherty and Hunt (2010).

**Figure 2:**
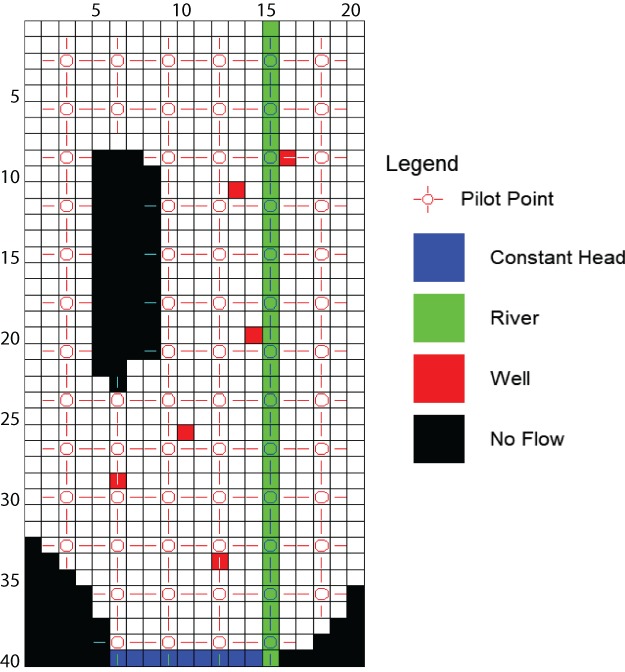
Location of pilot points used in place of the one *K* zone.

In this case, we introduced *K* parameter flexibility through 70 *K* pilot points (Doherty 2003; Doherty et al. 2010a) equally spaced over the model domain (one pilot point every three cells—Figure 2). The corresponding parameter estimation problem employed: (1) SVD, where the maximum number of singular values was set equal to the number of adjustable parameters and an eigenvalue ratio threshold for truncation of 5 × 10^−7^; and (2) Tikhonov regularization with 949 regularization equations to enforce a preferred homogeneity soft‐knowledge condition. Doherty and Hunt (2010) provided more detailed discussion of these settings and their effect on the parameter estimation.

Providing this additional parameter flexibility resulted in extremely well simulated observations (Table 6), which reflect the effectiveness of the methods employed as well as the fact that many of the problem characteristics were known and all Freyberg (1988) observations, and the additional flux observation, were error free. This is only expected in synthetic models—seeing such a good fit in an applied modeling effort suggests that the parameter estimation has overfit the (noisy) observations resulting in extreme parameter values (discussed below).

**Table 6:** Results of Pilot‐Point Model

a) Starting Heads Too High					
1 Zone starting *R* = 5.00 × 10^−4^					
70 Pilot points starting *K* = 1 m/d					
Base elevation = 3.34 fixed					
			**RMSE**	**RMSE**	
			**Cali-**	**Fore-**	**Number**
	***K***	***R***	**bration**	**casts**	**of**
	**(m/d)**	**(m/d)**	**(m)**	**(m)**	**of runs**
Mean	5.52	1.372 × 10^−4^	0.000	0.30	1305
Median	5.45				
Standard deviation	1.35				
Maximum	8.57				
Minimum	2.70				
Freyberg (1988)	2.6–10.4	1.380 × 10^−4^	0.06–0.37	1.26–7.96	15–38
**b) Starting Heads Too Low**					
1 Zone starting *R* = 1.30 × 10^−4^					
70 Pilot points starting *K* = 10 m/d					
Base elevation = 3.34 fixed					
			**RMSE**	**RMSE**	
			**Cali-**	**Fore-**	**Number**
	***K***	***R***	**bration**	**casts**	**of**
	**(m/d)**	**(m/d)**	**(m)**	**(m)**	**of runs**
Mean	5.52	1.372 × 10^−4^	0.000	0.30	1019
Median	5.45				
Standard deviation	1.35				
Maximum	8.57				
Minimum	2.70				
Freyberg (1988)	2.6–10.4	1.380 × 10^−4^	0.06–0.37	1.26–7.96	15–38

Note(s): Tikhonov regularization PHIMLIM = 1 × 10^−10^; results obtained using PEST v15.0 (R = recharge rate; K = horizontal hydraulic conductivity; RMSE = root mean squared error; m = meter; d = day).

**Figure 3:**
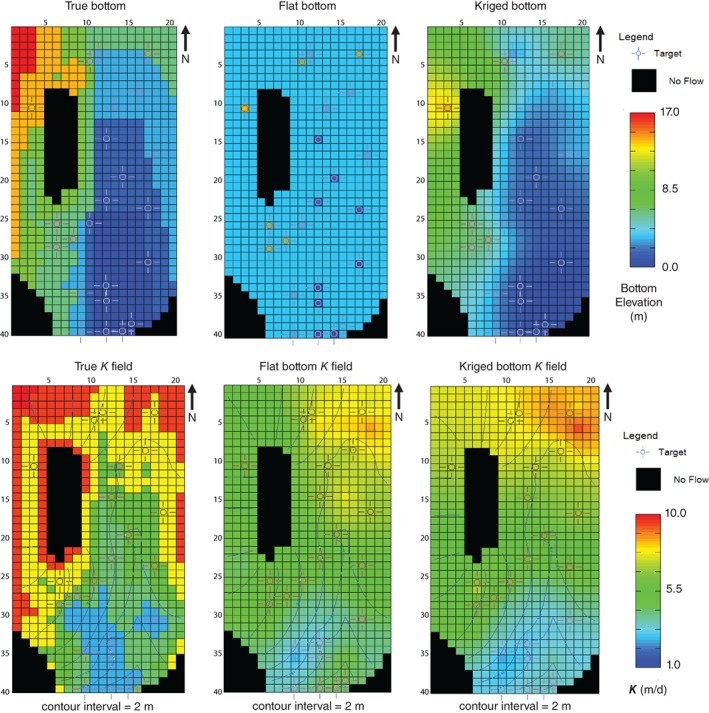
Comparison of the true, simplified flat, and simplified kriged bottom used for calibration is shown in the top row; *K* fields and water tables for the true model and those from pilot‐point model calibration of the flat and kriged bottom cases are shown on the bottom row. True base elevations were given to the students at target locations.

The results of the pilot point parameter estimation problem also demonstrate the effect of spatial parameter compensation in the resulting *K* field. Recall that a flat non‐varying base was assumed for parameter estimation purposes, yet the base of the true model rose in the northwest portion of the model domain (Figure 3). The parameter estimation uses the flexibility it is afforded—the *K* at the pilot points—to overcome the parameter simplification (or “structural”) error due to the overly simplified aquifer base. That is, the rising base elevation in the northwest reduced the saturated thickness, which reduced transmissivity; the model used a flat base in this area so the saturated thickness was too thick, which was then compensated by reducing *K* (to obtain a similar transmissivity). When a bottom elevation calculated from kriging the 16 elevations given the students was substituted for the flat bottom (Table 7), the calibrated *K* field began to look more like the true *K* field (bottom row, Figure 3). Areas without observations (e.g., the southwestern portion of the model), however, were not appreciably changed when the kriged bottom was added.

**Table 7:** Results of Pilot‐Point Model Calibration with a Flat Bottom Compared to a Kriged Bottom

a) Flat Bottom					
**Starting Heads Too High**					
1 Zone starting *R* = 5.00 × 10^−4^					
70 Pilot points starting *K* = 1 m/d					
Base elevation = 3.34 fixed					
			**RMSE**	**RMSE**	
			**Cali-**	**Fore-**	**Number**
	***K***	***R***	**bration**	**casts**	**of**
	**(m/d)**	**(m/d)**	**(m)**	**(m)**	**of runs**
Mean	5.52	1.372 × 10^−4^	0.000	0.30	1305
Median	5.45				
Standard deviation	1.35				
Maximum	8.57				
Minimum	2.70				
Freyberg (1988)	2.6–10.4	1.380 × 10^−4^	0.06–0.37	1.26–7.96	15–38
**b) Kriged Bottom**					
**Starting Heads Too High**					
1 Zone starting *R* = 5.00 × 10^−4^					
70 Pilot points starting *K* = 1 m/d					
Base elevation = variable (kriged) fixed					
			**RMSE**	**RMSE**	
			**Cali-**	**Fore-**	**Number**
	***K***	***R***	**bration**	**casts**	**of**
	**(m/d)**	**(m/d)**	**(m)**	**(m)**	**of runs**
Mean	5.66	1.367 × 10^−4^	0.000	0.29	1234
Median	5.70				
Standard deviation	1.73				
Maximum	9.35				
Minimum	2.60				
Freyberg (1988)	2.6–10.4	1.380 × 10^−4^	0.06–0.37	1.26–7.96	15–38

Note(s): Tikhonov regularization PHIMLIM = 1 × 10^−10^; results obtained using PEST v15.0 (R = recharge rate; K = horizontal hydraulic conductivity; RMSE = root mean squared error; m = meter; d = day).

Due to parameter compensation, the resulting optimal *K* values in the simplified flat‐bottomed model reflected not only their actual physical properties but also elements that offset the effects of simplifications made in other parts of the model (i.e., the incorrect base elevation).

As noted by Doherty and Welter (2010), optimal parameters from calibration reflect the information in the observations but also imperfections in the model such as those resulting from simplification. The resulting surrogate nature of an optimal parameter set can help or hinder the model's ability to make forecasts. Here a synthetic model “truth” was available that allowed the effect of the parameter compensation to be assessed directly as error in the parameter estimates and the forecasts. Small improvements in the forecasted heads at the pumping wells (Table 7) were obtained with the kriged bottom demonstrating that parameter surrogacy resulting from a flat bottom did not overly degrade the model's ability to make these forecasts. In real‐world applied modeling, it is not as easy to determine how parameter surrogacy affects model forecasts, and different forecast types will be affected to different degrees. However, strategies are available to minimize detrimental effects of model structural error (e.g., Doherty and Welter 2010; White et al. 2014). In any event, the pilot‐point parameterization yielded the best result of approaches tried (as evidenced by the lower forecast RMSE), and was clearly superior to the 1988 manual calibration.

### An Evaluation of Overfitting

**Figure 4:**
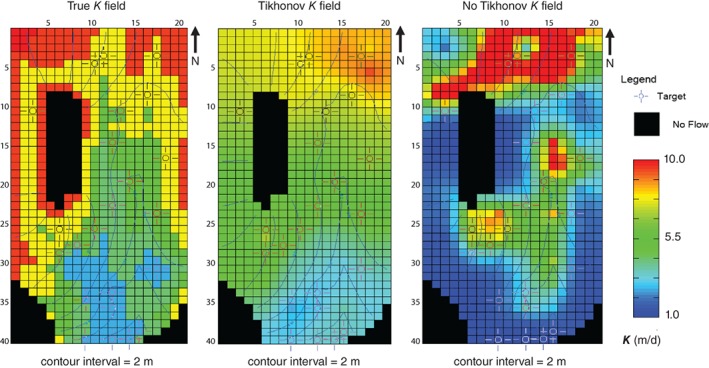
Comparison of the true *K* field (left) to those obtained using pilot points with (middle) and without Tikhonov regularization (right) using the kriged bottom model of Figure 3.

An important contribution of Freyberg (1988) was identifying and highlighting that a model that fits the observations best may not forecast best. This concern is of primary importance when calibrating highly parameterized models (especially those using pilot points). The highly parameterized approach often achieves an excellent fit but can also “overfit,” where the parameter estimation chases noise in the observations and yields unrealistic parameter values and distributions (e.g., parameter “bullseyes,” or hotspots). Tikhonov regularization addresses overfitting by balancing fit with deviations from the modeler's soft‐knowledge preferred conditions for parameters. Here, a target measurement objective function (PEST variable PHIMLIM—see Fienen et al. (2009) for details) of 1 × 10^−10^ was used to focus on fit to the error‐free observations. A test was conducted using the kriged bottom model of Table 7 where Tikhonov regularization was omitted. The maximum number of singular values was again set equal to the number of adjustable parameters with an eigenvalue threshold of 5 × 10^−7^; thus, little to no smoothing of the parameter field was possible by SVD truncation. Omitting Tikhonov regularization yielded parameter values higher and lower than the true range (whereas optimal *K* values reported in Tables 4, 5, 6, 7 fell within the range of true *K* values). Worse yet, forecast accuracy was degraded (forecast RMSE, Table 8), and the distribution contained parameter hotspots (Figure 4). The fit to the observations was indeed better (i.e., reduced measurement objective function in Table 8) but required unreasonable parameter values for a small improvement in fit.

**Table 8:** Results of Pilot‐Point Model Calibration with and Without Tikhonov Regularization

a) Tikhonov Regularization (PHIMLIM = 1 × 10^−10^)					
**Starting Heads Too High**					
1 Zone starting *R* = 5.00 × 10^−4^					
70 Pilot points starting *K* = 1 m/d					
Base elevation = variable (kriged) fixed					
Optimal Measurement objective function = 3.812 × 10^−8^					
			**RMSE**	**RMSE**	
			**Cali-**	**Fore-**	**Number**
	***K***	***R***	**bration**	**casts**	**of**
	**(m/d)**	**(m/d)**	**(m)**	**(m)**	**of runs**
Mean	5.66	1.367 × 10^−4^	0.000	0.29	1234
Median	5.70				
Standard deviation	1.73				
Maximum	9.35				
Minimum	2.60				
Freyberg (1988)	2.6–10.4	1.380 × 10^−4^	0.06–0.37	1.26–7.96	15–38
**b) No Tikhonov Regularization**					
**Starting Heads Too High**					
1 Zone starting *R* = 5.00 × 10^−4^					
70 Pilot points starting *K* = 1 m/d					
Base elevation = variable (kriged) fixed					
Optimal Measurement objective function = 5.907 × 10^−11^					
			**RMSE**	**RMSE**	
			**Cali-**	**Fore-**	**Number**
	***K***	***R***	**bration**	**casts**	**of**
	**(m/d)**	**(m/d)**	**(m)**	**(m)**	**of runs**
Mean	5.24	1.341 × 10^−4^	0.000	1.32	1221
Median	2.80				
Standard deviation	6.45				
Maximum	32.54				
Minimum	0.94				
Freyberg (1988)	2.6–10.4	1.380 × 10^−4^	0.06–0.37	1.26–7.96	15–38

Note(s): Results obtained using PEST v15.0 (R = recharge rate; K = horizontal hydraulic conductivity; RMSE = root mean squared error; m = meter; d = day).

If observations were error free so that fitting to measurement uncertainty was impossible, what is the source of overfitting? Recall that error in observations is only one source of error during parameter estimation; parameter simplification errors also result from the structure chosen for the model (e.g., Gaganis and Smith 2001). In this case, use of a flat or kriged bottom elevation array results in a structural error. The parameter estimation uses the flexibility afforded the pilot points to mitigate the structural error and explores parameter values outside of those needed when the true bottom elevation was used. The potential for overfitting—shown here to be possible even if observations were error free—underscores the importance of reserving the term “calibration” for models that have both acceptable fit *and* reasonable parameter values and distributions (Chapter 9—Anderson et al. 2015). Such a focus becomes even more important in applied modeling as all observations have inherent uncertainty and structural error is pervasive, which increases the potential for overfitting. Introducing error to the Freyberg (1988) observations and further exploration of the overfitting problem is considered in more detail in future companion work.

### An Evaluation of Head Interpolation Effects

The 1988 students likely processed the MODFLOW head output by manually extracting cell‐centered heads, resulting in discontinuous heads at the node boundaries. Nowadays, MODFLOW output is typically interpolated to avoid this discontinuity. Models discussed thus far were generated with a graphical user interface, which post‐processed MODFLOW output using head interpolation. To test the effect of this interpolation, the kriged bottom pilot‐point model of Table 7 was modified to use non‐interpolated, MODFLOW cell‐center heads in the parameter estimation process. For this reconstructed model, not interpolating heads only slightly degraded the model's ability to forecast heads in the pumping wells but required 153 (12%) more additional forward runs for parameter estimation convergence (Table 9). Artifacts resulting from using grids to simulate heads near pumping wells are well recognized (e.g., pages 259‐269 in Anderson et al. 2015); thus, non‐interpolation artifacts are expected for pumping well forecasts. The difference in number of runs required likely relates to a smoother head surface being represented with more internal numerical precision, which helps the derivative‐based nonlinear regression methods find a best fit. For this problem the difference in total parameter estimation runtime is small because the model has a short forward runtime (<1 min); beneficial effects of interpolation are expected to be larger in models with larger grid cells, larger horizontal gradients, or long forward‐run times. However, further study is needed to understand ramifications of spatial interpolation over a range of forecasts using imperfect real‐world models.

**Table 9:** Results of Pilot‐Point Model Calibration with Interpolated and Non‐Interpolated Head Output

a) Interpolated Head Post‐Processing					
**Starting Heads Too High**					
1 Zone starting *R* = 5.00 × 10^−4^					
70 Pilot points starting *K* = 1 m/d					
Base elevation = variable (kriged) fixed					
			**RMSE**	**RMSE**	
			**Cali-**	**Fore-**	**Number**
	***K***	***R***	**bration**	**casts**	**of**
	**(m/d)**	**(m/d)**	**(m)**	**(m)**	**of runs**
Mean	5.66	1.367 × 10^−4^	0.000	0.29	1234
Median	5.70				
Standard deviation	1.73				
Maximum	9.35				
Minimum	2.60				
**b) Non‐Interpolated Head Post‐Processing**					
**Starting Heads Too High**					
1 Zone starting *R* = 5.00 × 10^−4^					
70 Pilot points starting *K* = 1 m/d					
Base elevation = variable (kriged) fixed					
			**RMSE**	**RMSE**	
			**Cali-**	**Fore-**	**Number**
	***K***	***R***	**bration**	**casts**	**of**
	**(m/d)**	**(m/d)**	**(m)**	**(m)**	**of runs**
Mean	5.66	1.367 × 10^−4^	0.000	0.31	1387
Median	5.70				
Standard deviation	1.73				
Maximum	9.35				
Minimum	2.60				

Note(s): Tikhonov regularization PHIMLIM = 1 × 10^−10^; results obtained using PEST v15.0 (R = recharge rate; K = horizontal hydraulic conductivity; RMSE = root mean squared error; m = meter; d = day).

### Including Forecast Uncertainty Analysis

Most applied groundwater models are constructed to simulate forecasts of interest (Anderson et al. 2015). As a result, the ability of a model to accurately forecast is typically more important than its ability to reproduce what has already been measured (calibration observation data). Or put another way: today's groundwater modeling efforts should put “forecasts first” (White 2017). With such an approach, forecasts are included in the earliest models constructed, and effects of model changes are evaluated for both forecasts and observations used for calibration. All forecasts have uncertainty, thus focusing on changes to forecast uncertainty at all phases of modeling broadens the modeler's analysis beyond model fit. This forecast‐uncertainty focus facilitates insight regarding how robust model forecasts are throughout the calibration process. Moreover, such insight can have more utility than parameter sensitivity analyses performed after calibration (Anderson et al. 2015).

Freyberg (1988) also recognized the distinction between calibration and forecast accuracy and emphasized forecasted heads in the pumping well. However, the subjective nature of manual trial‐and‐error calibration did not provide a quantitative framework to formalize forecast uncertainty analyses. The parameter estimation framework used here, however, does. Evaluating uncertainty approaches is the subject of our companion work; here we focus on the computationally frugal “first‐order, second moment,” or “linear” uncertainty analysis available in PEST++; these techniques are also available using PEST utilities (PREDUNC‐ Doherty et al. 2010b) and pyEMU (White et al. 2016). These techniques can be framed in a Bayesian context as they start with a prior estimate of uncertainty in the parameter values (typically supplied using parameter bounds or using geostatistics). Next, a Bayesian update was performed using the calibration process as the new information, which resulted in a posterior estimate of uncertainty that reflects both the prior estimate and the information gleaned from calibration (see Fienen et al. 2010 for additional discussion of the Bayesian context). To access this capability in PEST++, the modeler simply adds a line to the PEST++ control file identifying which model outputs are forecasts. PEST++ outputs the standard deviation before calibration (prior) and after calibration (post) for each forecast as well as the 95% confidence interval. For discussion purposes, a successful calibration/uncertainty exercise are those where the true forecast value lies within the posterior 95% confidence interval. Because linearity is assumed, uncertainty reported is normally distributed around each forecasted value.

**Figure 5:**
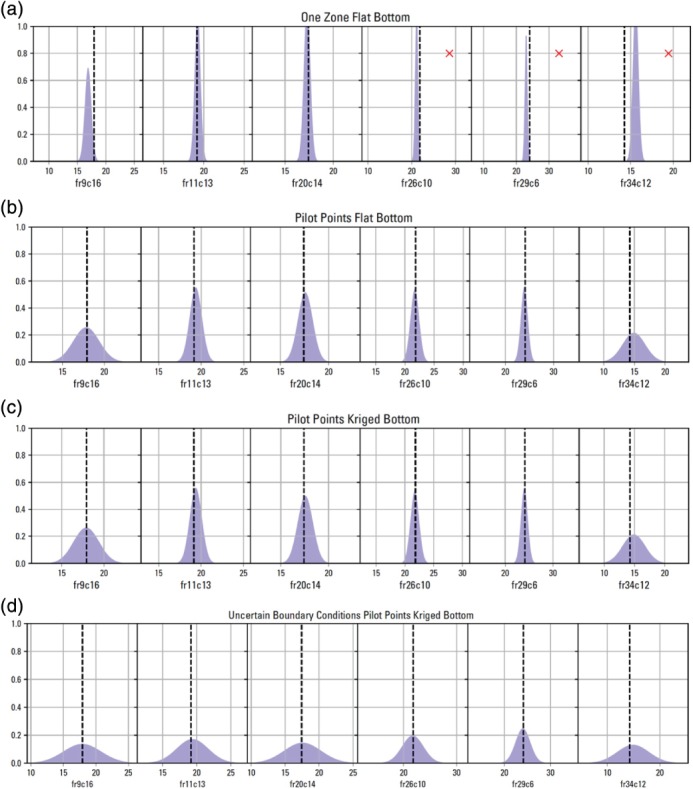
True forecasts of heads in pumping wells (vertical dotted line) and probability calculated using linear uncertainty analysis (a red “x” indicates the true forecast is outside of the 95% confidence interval; forecast names indicate row and column of each forecast). Y‐axes represent probability density.

When evaluated for the parameter estimation runs described above, half of the 1‐zone model forecasts fell outside the posterior 95% confidence interval (Figure 5a). However, this is not necessarily due to poor model simulation; rather, it is because the error bounds around the forecast are small. The narrow uncertainty bounds are an artifact of parameter simplification error of the 1‐zone model not being properly accounted for within the uncertainty analysis. Parameters not evaluated during the uncertainty analysis were considered perfectly known, thereby contributing nothing to uncertainty (Hunt 2017). Put another way: assuming a single *K* zone ignored uncertainty imparted to the forecasts from the “missing” *K* parameters—rather, it is telling the uncertainty analysis that we are certain that the system is homogeneous throughout the model domain. This can be further extended to applied modeling in general: anything not parameterized (and therefore adjusted during parameter estimation) is implicitly treated as perfectly known in uncertainty analyses. This is also true for *R* and pumping rates for future conditions, which are not informed by calibration, but may strongly influence the forecasts of interest.

Performance of the highly parameterized model was demonstrably better, with all forecasts falling within the range of reported uncertainty (Figure 5b and 5c). This suggests that increased parameter flexibility, and resulting type and degree of parameter surrogacy, improves forecasting. However, recall that the performance of the uncertainty analysis is a direct result of information gained during calibration. Because observations and many properties of the model were error‐free, the signal contained in the information gained during calibration was maximized. Therefore, the model was well‐suited for making these types of forecasts of interest. Other forecasts, such as travel time and path, may not be as well served by the parameter surrogacy resulting from model simplification.

A last uncertainty analysis illustrates a more typical applied modeling effort where boundary conditions—namely bottom elevation, river conductance, pumping rates, and specified heads—are not known perfectly (Figure 5d). The effect of removing certainty in the assumed boundary condition is apparent as the distributions are now wider and shorter (i.e., more uncertain). Figure 5d evaluated only a subset of the possible model boundary conditions; most applied models also include uncertainty in all boundary conditions as well as in the observations. Indeed, all models of an unknowably complex natural system will be ill‐posed and underdetermined, thus will not have a unique solution to the inverse problem. Therefore, multiple parameter sets will provide similar model fits, but may also provide appreciably different forecasts. This source of forecast uncertainty is only partly addressed by obtaining a unique solution to the inverse problem in the ways described above. More comprehensive uncertainty analyses recognize that multiple parameter sets, and in some cases multiple conceptual models, need to be explored for more expansive reporting of forecast uncertainty. Our companion work expands on this “forecast first” approach and explores these considerations with a more realistic, and uncertain, construction of the Freyberg (1988) model.

### How Do Parameter Estimation Approaches Perform Compared to the 1988 Effort?

We have focused here on the root mean square error (RMSE) of forecasted heads in the pumping wells after the river was lined, however this is but one of three 1988 metrics used to judge the performance of the student groups. The other two metrics were the number of model runs and the RMSE from the true *K* field for the 705 active nodes. Performance across all three metrics was summarized in a stacked bar chart (Figure 6 in Freyberg 1988). Each metric was normalized using the best and worst result. For example, for the number of runs metric, the contribution to the bar chart was:

(1) $$\mathrm{Bar}\;\text{Height}=0.33\left(\frac{N_{\max}-N}{N_{\max}-N_{\min}}\right)$$

where *N* represents the number of runs for a given student group*, N*_max_ the most runs for all groups, and *N*_min_ the fewest runs for all groups. Thus, the best effort with the fewest runs would result in a value of 0.33 and the worst effort with the most runs would be given a 0.0; the highest possible performance across all three metrics was 0.99.

**Figure 6:**
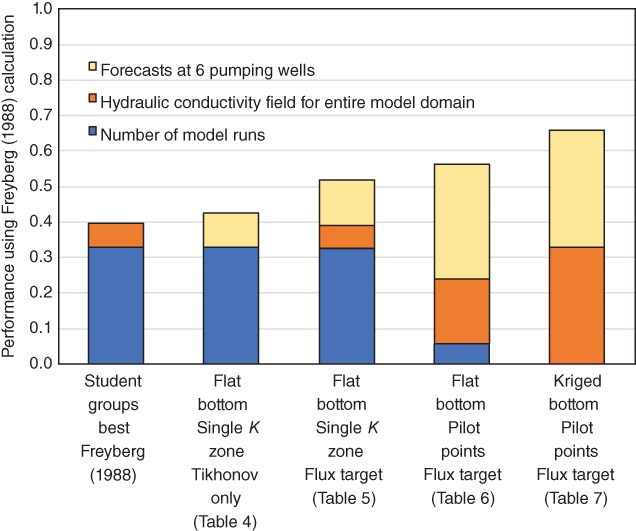
Evaluation of model performance, where higher bars reflect better performance. Highest performance possible is 0.99.

We applied these performance metrics to the parameter estimation approaches discussed here (Figure 6). For comparison, the best result for each of the three metrics reported in Freyberg (1988) was used for comparison regardless of student group, so the 1988 bar reflects the best (combined) case for the entire manual calibration exercise. In all cases the automated and systematic approaches to parameter estimation outperformed the combined best reported manual exercise, even when accounting for the increased number of runs for the highly parameterized case. However, recall that the original 1988 problem was ill‐posed because it lacked a flux observation. A flux target was needed to obtain the three rightmost bars in Figure 6.

## Where We Have Come in 30 Years

This work formalizes and supports many of the conclusions of Freyberg (1988). Consider the following:

- In 1988 it was concluded that the results “clearly demonstrate the non‐uniqueness of solutions to an inverse problem.” Strictly speaking this would be difficult to support when testing consisted of only manual trial‐and‐error calibration that terminated subjectively. However, parameter estimation software was in its infancy at the time and alternatives to manual trial‐and‐error were not widely used. The quantitative framework of today's software unambiguously supports Freyberg's conclusion—the 1988 problem did not have a unique solution when traditional approaches for mathematically well‐posed and overdetermined problems were used. However, including soft knowledge and using modern approaches designed for ill‐posed/underdetermined problems yielded a conditionally unique solution. Notably, the predictive power of that model was superior to even the best manually calibrated forecasts.
- Freyberg (1988) observed that “good calibration…does not equal good prediction” (page 360). The results here show the very best fit model (obtained by excluding Tikhonov and SVD truncation regularization—Table 8) had the parameter estimation process chasing machine and structural noise. The result was an overfit *K* field (unreasonable/extreme parameter values) and decreased forecasting accuracy. The model that employed soft‐knowledge through Tikhonov regularization provided the best forecasts.
- Freyberg (1988) highlighted something that is now well accepted: “The goal of parameter identification is rarely the parameter estimates. Rather, the ultimate goal is nearly always defined by a prediction problem” (page 354). Observed data used for calibration tell us what we have already experienced and measured. Because a model fits what we already know does not necessarily mean it yields representative forecasts for what we do not know. Rather, modeling objectives usually involve forecasts of the system under conditions different from those experienced during site characterization. Therefore, applied modeling will be more successful in a “forecasts first” paradigm.

Notable updates to the state of the science have also occurred since 1988. These include:

- Parameter correlation between *K* and *R* cannot be overcome using only head data for calibration. These results underscore the appropriateness of the Anderson et al. (2015) requirement of both head and flux target types.
- Perhaps most importantly: a central conclusion of Freyberg (1988) was that “the group achieving the best prediction chose to zone the conductivity field into a relatively few homogeneous regions, while the group producing the worst prediction chose to ‘tweak’ the conductivity field grid block by grid block to achieve a good (in fact, the best) local fit to the observed data.” In the work here, the opposite was demonstrated when parameter estimation was applied: the most highly parameterized model, when properly implemented with soft knowledge, performed the best for both calibration and forecasting. The flexibility provided by the larger number of parameters allowed the information from observations to flow more effectively, and in appropriate ways, to the parameters that can be informed by it, which in turn reduced the model's structural error and increased its ability to forecast. Yet, when soft knowledge was not used, the highly parameterized model yielded parameter estimates that were overfit with respect to structural noise, resulting in extreme parameter values and worse forecasts. *Therefore, it is not the number of parameters that cause poor performance but how those parameters are handled*. The superior performance of the highly parameterized model should give pause to those using statistics that automatically weight models with fewer parameters superior to those with more parameters.
- Qualitatively judging the value of a calibration exercise by the number of forward model runs is not particularly meaningful today. Indeed, the best results obtained here involved more forward model runs than any group in the 1988 exercise. One might argue that for any manual calibration effort, a “number‐of‐runs” criterion will say more about the tenacity (and perhaps insightfulness and inquisitiveness) of the modelers than the model's ability to provide a high‐quality forecast. More to the point, the computing resources available to today's modelers are unprecedented. High throughput parallel computing (e.g., Fienen and Hunt 2015) and cloud computing (e.g., Hunt et al. 2010) allow parameter estimation of highly parameterized problems (i.e., parameters numbering in the thousands or more). Of course, such parallel computing resources were not needed for any of the results shown here—each parameter estimation was run on a single desktop computer in less than 20 min. Regardless, there should be no expectation that model forecasts will be improved by reducing the number of model runs.
- Finally, reporting uncertainty in model forecasts is expected more today than in 1988. As highlighted by many (e.g., Hester and Coleman 2014; Anderson et al. 2015; White 2017; Hunt 2017—among many others), decision making is best informed when representative uncertainty estimates are provided. The students in 1988 could not quantitatively address uncertainty because the subjective trial‐and‐error process used does not have a quantitative framework from which to construct an uncertainty analysis. However, because the model is synthetic and “truth” is known, the quantitative parameter‐estimation framework can assess how well a simple linear uncertainty method captures the model's ability to make representative forecasts. When the forecast uncertainty was evaluated using a computationally frugal linear analysis, the 1‐zone simple model performed the worst (half of the forecasts fell within the uncertainty bound) and the highly parameterized model performed best (all forecasts within reported uncertainty bounds).

This last point—the focus on uncertainty analysis—motivated additional analysis and further extension of the Freyberg (1988) model that is the subject of ongoing work, where the intent is to provide the reader with tools to replicate, test, and explore our results. In this way we hope to truly carry on the spirit of Freyberg (1988) into today's applied modeling.

## Authors' Note

The author(s) does not have any conflicts of interest or financial disclosures to report. Any use of trade, firm, or product names is for descriptive purposes only and does not imply endorsement by the U.S. Government.
